# Organic Solvent Tolerant Lipases and Applications

**DOI:** 10.1155/2014/625258

**Published:** 2014-02-02

**Authors:** Shivika Sharma, Shamsher S. Kanwar

**Affiliations:** Department of Biotechnology, Himachal Pradesh University, Summer Hill, Shimla 171 005, India

## Abstract

Lipases are a group of enzymes naturally endowed with the property of performing reactions in aqueous as well as organic solvents. The esterification reactions using lipase(s) could be performed in water-restricted organic media as organic solvent(s) not only improve(s) the solubility of substrate and reactant in reaction mixture but also permit(s) the reaction in the reverse direction, and often it is easy to recover the product in organic phase in two-phase equilibrium systems. The use of organic solvent tolerant lipase in organic media has exhibited many advantages: increased activity and stability, regiospecificity and stereoselectivity, higher solubility of substrate, ease of products recovery, and ability to shift the reaction equilibrium toward synthetic direction. Therefore the search for organic solvent tolerant enzymes has been an extensive area of research. A variety of fatty acid esters are now being produced commercially using immobilized lipase in nonaqueous solvents. This review describes the organic tolerance and industrial application of lipases. The main emphasis is to study the nature of organic solvent tolerant lipases. Also, the potential industrial applications that make lipases the biocatalysts of choice for the present and future have been presented.

## 1. Introduction

**Figure 1:**
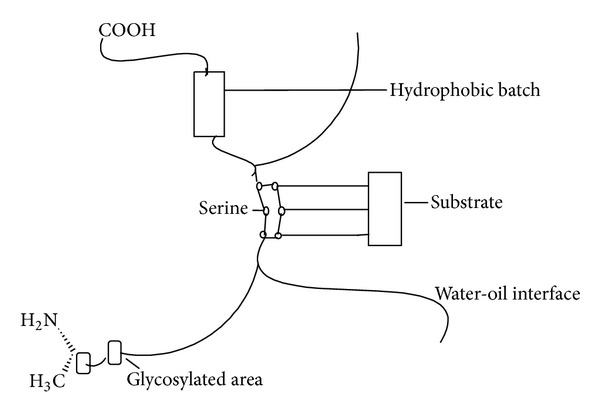
Diagrammatic representation of a lipase molecule showing its main features.

**Figure 2:**
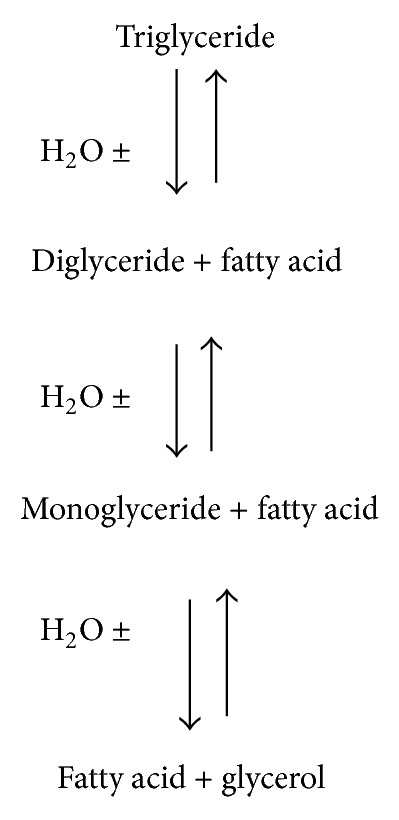
The mediated lipase reaction(s).

Lipases have emerged as one of the leading biocatalysts with proven potential for contributing to the multibillion dollar underexploited lipid bioindustry. They have been used in *in situ *lipid metabolism and *ex situ *multifaceted industrial application(s) [1] and the catalyze the hydrolysis of triacylglycerol to glycerol and fatty acids [2]. Lipases find potential applications in bioprocesses largely due to their availability and stability in organic as well as in aqueous media [3–5]. This enzyme has versatile applications by virtue of its unique properties [6, 7]. Under natural conditions, lipases catalyze the hydrolysis of ester bonds at the interface between an insoluble substrate phase and the aqueous phase where the enzyme remains dissolved (Figure 1). In nonaqueous conditions they catalyze the reverse reaction (such as esterification, interesterification, and transesterification) producing glycerides (Figure 2) from glycerol and fatty acids [8, 9]. In the past years, a better understanding of enzymes functionalities and catalytic behaviors, together with the progress of molecular engineering, has led to new applications for various types of enzymes as, for example, proteases, acylases, oxidases, amylases, glycosidases, cellulases, or lipases. Lipases have improved substrate specificity and operate in milder reaction conditions. Moreover, the fact that they retain their activity in organic solvents and also their catalytic promiscuity extend their range of applications [10, 11].

Lipases are ubiquitous in nature and are produced by various plants, animals, and microorganisms. However, for the production of industrial enzymes, microorganisms are the most preferred source. They have the shortest generation time, high yield of conversion of substrate into product, great versatility to adapt to environmental conditions, and simplicity in genetic manipulation as well as in cultivation conditions [12]. Microbial lipases are currently receiving much attention with the rapid development of enzyme technology [13] and due to their ability to perform catalysis at extremes of temperature, pH, and organic solvents with chemo-, regio-, and enantioselectivity. Also lipases display useful properties related to their stability as organic solvent-tolerant [14] and thermostable [15] enzymes. The reason for the enormous biotechnological potential of microbial lipases includes the fact that they are stable in organic solvents, do not require cofactors, and possess a broad specificity [16].

Fundamental studies on polymerization revealed some remarkable capabilities of lipases for polymerization chemistry. The polymerization and transesterification studies generally demand harsh condition(s) of presence of organic solvents and/or high temperature [17, 18]. The technological utility of enzymes can be enhanced greatly by using them in organic solvents rather than their natural aqueous reaction media [19]. The use of enzyme in organic media has exhibited many advantages: increased activity and stability, regiospecificity and stereoselectivity, higher solubility of substrate, ease of products recovery, and ability to shift the reaction equilibrium toward synthetic direction [20]. From the biotechnological point of view, there are numerous advantages of conducting immobilized biocatalyst-promoted enzymatic conversions in organic solvents as opposed to reactions performed in water-based system(s) [21].

## 2. Sources of Lipases

Lipases are produced by plants, animals, and microbes but only microbial lipases are found to be industrially important since they are diversified in their enzymatic properties and substrate specificity [22]. Lipases can be obtained from animals mainly from fore-stomach tissue of calves or lambs and pancreatic tissues of pigs. The disadvantages of using animal lipases include presence of trypsin in pig pancreatic lipases, which results in bitter tasting amino acids, and presence of residual animal hormones or viruses as well as their undesirable effects in the processing of vegetarian or kosher diets [23]. Plant lipases are also available but not exploited commercially because of the yield and the processes involved. Thus, microbial lipases are currently receiving more attention because of their technical and economic advantages, where the organisms are cultivated in medium containing appropriate nutrient composition under controlled conditions. Also, lipase production by microorganisms varies according to the strains, the composition of the growth medium, cultivation conditions, pH, temperature, and the kind of carbon and nitrogen sources [24, 25]. Generally, bacteria, fungi, yeast, and actinomycetes are recognized as preferred sources of extracellular lipases, facilitating the enzyme recovery from the culture broth, although *Candida, Pseudomonas, Mucor, Rhizopus, *and *Geotrichum *spp. stand out as the major commercially viable strains [26]. Bacterial lipases are extensively used in food industry for quality improvement, dairy industry for hydrolysis of milk fat, cheese ripening, beverages to improve aroma, and health foods for transesterification [6]. Many microorganisms have been reported in the last decade for lipase production in both submerged and solid-state fermentations reported (Table 1).

## 3. Reactions Catalyzed by Lipases

The lipases catalyze a wide range of reactions, including hydrolysis, interesterification, alcoholysis, acidolysis, esterification, and aminolysis. They catalyse the hydrolysis of fatty acid ester bond in the triacylglycerol and release free fatty acids. The lipase-catalyzed reactions are:

(i) *Hydrolysis*
  (1) $$\begin{array}{c} {\text{RCOOR}}^{\prime }+{\text{H}}_{2}\text{O}⟶\text{RCOOH}+{\text{R}}^{\prime }\text{OH} \end{array}$$
(ii) *Synthesis.* Reactions under this category can be further separated into the following categories.
  (a) *Esterification*
    (2) $$\begin{array}{c} \text{RCOOH}+{\text{R}}^{\prime }\text{OH}⟶{\text{RCOOR}}^{\prime }+{\text{H}}_{2}\text{O} \end{array}$$
  (b) *Interesterification*
    (3) $$\begin{array}{c} {\text{RCOOR}}^{\prime }+{\text{R}}^{\prime \prime }\text{COO}{\text{R}}^{*}⟶\text{RCOO}{\text{R}}^{*}+{\text{R}}^{\prime \prime }\text{COO}{\text{R}}^{\prime } \end{array}$$
  (c) *Alcoholysis*
    (4) $$\begin{array}{c} {\text{RCOOR}}^{\prime }+\;{\text{R}}^{\prime \prime }\text{OH}⟶\text{RCOO}{\text{R}}^{\prime \prime }\;+\;{\text{R}}^{\prime }\text{OH} \end{array}$$
  (d) *Acidolysis*
    (5) $$\begin{array}{c} {\text{RCOOR}}^{\prime }+{\text{R}}^{\prime \prime }\text{COOH}⟶{\text{R}}^{\prime \prime }\text{COO}{\text{R}}^{\prime }+\text{RCOOH} \end{array}$$

The last three reactions are often grouped together into a single term, namely, transesterification. The term transesterification refers to the exchange of groups between an ester and an acid (acidolysis), between an ester and an alcohol (alcoholysis), or between two esters (interesterification). The ability of lipases to catalyze these reactions with great efficiency, stability, and versatility makes these enzymes highly attractive from a commercial point of view. The lipase specificities can be divided [27, 28] into three main groups as follows.

*(1) Substrate Specificity.* The natural substrates are glycerol esters. These enzymes are able to catalyze the hydrolysis not only of triacylglycerols (TAGs), but also di- and monoacylglycerols and even phospholipids, in the case of phospholipases.

*(2) Regioselective.* Regioselectivity is the preference of one direction of chemical bond making or breaking over all other possible directions. It is subdivided into the following types.

- Nonspecific lipases: they catalyze the complete hydrolysis of triacylglycerols into fatty acids and glycerol in a random way; monoglycerols and diacylglycerols are the intermediate products.
- Specific 1.3 lipases: they only hydrolyse triacylglycerols at the C1 and C3 glycerol bonds, producing fatty acids, 2-monoacylglycerols, and 1,2-diacylglycerols or 2,3-diacylglycerols, the latter two being chemically unstable, promoting migration of the acyl group producing 1,3-diacylglycerol and 1-monoacylglycerols or 3-monoacylglycerols.
- Specific or selective type fatty acid: lipases can be specific for a particular type of fatty acid, or more frequently, for a specific group of fatty acids. They hydrolyze fatty acid esters located at any triacylglycerol position.

*(3) Enantioselective.* Lipases have the ability to discriminate enantiomers in a racemic mixture. An example of this is the R-isomer of Aspartame, which tastes sweet, whereas the S-isomer tastes bitter. The enantiospecificities of lipases can vary according to the substrate and this variation can be connected to the chemical nature of the ester [29].

## 4. Lipase Behavior/Properties in Organic Solvents

### 4.1. Lipase Structure in Organic Solvents

Molecular modeling of *R. miehei *lipase in different environments showed that the structure modeled in vacuum was reasonably similar to the models obtained with hydrophobic solvents as the environment [30]. Generally, hydrophilic solvents have relatively more interactions with the enzyme molecules. The structure of an enzyme, Subtilisin Carlsberg, in a hydrophilic solvent, acetonitrile, which was determined by X-ray crystallography, was shown to be similar to that in water [31]. The water bound to the enzyme molecules, however, decreased and acetonitrile molecules were bound to the enzyme molecules. Sequences for several lipases and esterases were compared to find the residues that are either completely or partially conserved [32]. Sequence alignment of 16 lipoprotein lipases showed a large area of sequence identity in the proximity of the active-site serine residue. Moreover, all the lipases possessed a G∗S∗G motif around the active-site serine. The sequences of these lipases showed a close relation with sequences of hepatic lipases, which were less closely related to sequences of pancreatic lipases. The parts of lipases that are conserved may be important for their structural integrity, activity, and/or specificity [32].

### 4.2. Lipase Stability in Organic Media

A majority of enzymes show good *in vitro *catalytic rates in aqueous solutions. However, lipases, being activated by interfaces, show lower catalytic rates in homogeneous aqueous solutions than in the presence of interfaces, for example, water-organic solvent interface [33]. Typically, lipases are ubiquitous enzymes that were originally characterized by their ability to catalyze the hydrolysis of acylglycerides, fatty acid esters, and so forth at oil-water interface [34]. This is called aqueous-organic solvent biphasic system. Vigorous mixing of the two phases forms a suspension with a significantly large interfacial area.

Enzyme molecules are solubilized in discrete hydrated reverse micelles formed by surfactants, within a continuous phase of a nonpolar organic solvent, that is, in reverse micellar system. Under appropriate conditions, a reverse micellar solution is homogeneous, thermodynamically stable, and optically transparent [35]. The applications of enzymes in organic media rather than aqueous media have several important advantages such as the shift in thermodynamic equilibrium in favour of the product over the hydrolytic reaction, an increased solubility of nonpolar substrates, elimination of side reactions, and an increased thermal stability of the enzyme in harsh conditions [21]. Most lipases are known to be active and stable in anhydrous organic solvents. Several covalent modifications as well as noncovalent modification techniques have been developed for solubilization of lipases in organic solvents [36]. The biological origin of lipase, the reaction to be carried out (hydrolysis or esterification), the substrates used, and so forth determine which solvent system will be the most suitable one. The use of organic solvents in reaction media shifts the thermodynamic equilibrium to favor synthesis over hydrolysis. Furthermore, in organic solvent the conformation of the enzyme appears to be more rigid. These characteristics enable controlling some of the enzyme's catalytic properties, such as the substrate specificity, the chemo, region-, and enantioselectivity by variation of the solvent [37]. Recently, lipases from *Pseudomonas* are the most widely used in biotechnological application because of their potential in organic synthesis for highly valuable chemicals [38]. Also, the cost of downstream processing is a direct function of the solvent system and the impurities present. Potential advantages (Table 2) of employing enzymes in nonaqueous media as opposed to aqueous media were postulated by many authors [39–41].

Lipase can undergo deactivation in the synthetic reactions due to altered temperature, shear stresses, exposure to interfaces, and chemical denaturants, which are generally present in the esterification reaction systems as either substrates or products. This enzyme deactivation occurs either due to physical changes in the enzyme structure or chemical changes like deamidation and breakage of disulfide bonds. The esterification reactions are always carried out in nonaqueous solvents. The stability of enzymes in organic solvents is a strong function of the solvent properties.

Deactivation of a lipase from *R. miehei *due to temperature and butanol has been studied [42] and a considerable deactivation of the enzyme by butanol was noticed. Thermal deactivation of enzymes usually occurs due to unfolding of the molecule. At high temperature, various forces maintaining the enzyme structure (including hydrogen-bonding, ionic and van der Wall interactions, and hydrophobic interactions) diminish, leading to unfolding of the enzyme. Thermal deactivation of lipase may be reduced considerably by its immobilization. Thermal deactivation of lipase B from *Candida antarctica *(CALB) and lipase from *Candida rugosa *(CRL), respectively, in their native and immobilized forms has been studied [43, 44]. Lyophilization of the enzyme together with certain additives such as carbohydrates [45, 46], fatty acids [47] or salts [48], has been shown to greatly improve the enzyme performance. The activating effect of additives is more pronounced in dry organic solvents than in partially hydrated organic solvents [49]. The treatment of *Candida rugosa *lipase with short-chain polar organic solvents (methanol, ethanol, 1- and 2-propanol, 1- and 2-butanol, or/and acetone) enhanced its esterification and transesterification activity [50]. *F. oxysporum* has an increase of its activity after being incubated in organic solvents, which is an essential feature in many organic syntheses [37].

In organic media, the pH dependence of enzymes dispersed in a solvent has been shown similar to the pH dependence of the enzymes in an aqueous medium [51, 52]. The dependence of the enzyme activity in organic solvents on pH of the aqueous solution in which the enzyme last existed is termed pH memory. The ionization states of lyophilized compounds are similar to those in solution form in which the compound was lyophilized [53]. Although this latest investigation of lyophilized compounds using Fourier transform infrared (FTIR) spectroscopy supports the concept of pH memory, there are exceptions to this concept.

### 4.3. Solubilization of Lipases in Organic Solvents

Lipases are insoluble in organic solvents in their native form. Solubilized lipases are attractive not only because of their higher activities (compared to insoluble lipases) but also because of their optical transparency that allows one to perform structural characterization by spectroscopic technique [54]. Two types of methods, namely, covalent and noncovalent modifications, are in common use for lipase solubilization. Chemical or covalent modifications are done by using chemical modifiers like polyethylene glycol (PEG), poly-*N*-vinylpyrrolidone, polystyrene, polymethyl methacrylate [55, 56], and nitrocellulose membrane [57]. Such chemical modifications greatly affect the activity, stability, and selectivity of the enzyme [58, 59] as well as its reusability [57].

Noncovalent modification of lipases has essentially been restricted to coating the lipase molecule with different surfactants. One of the most widely reported techniques involves dissolution of the lipase and the surfactant in aqueous solution [60, 61]. When sufficient time is allowed, the hydrophilic tails of the surfactant noncovalently bind to polar/ionic groups on the surface of lipase, thereby creating an enzyme-surfactant complex whose surface is hydrophobic because of the protruding hydrophobic tails of the surfactant. This enzyme-surfactant complex, which precipitates from the aqueous solution, can be dissolved in organic solvents. The solubilized lipase has shown catalytic activities, which are significantly greater than those demonstrated by lipase powders. Another related technique involves preparation of water-in-oil emulsions (or reverse micelles) containing lipase and then drying out the water from the emulsion phase that yields a lipase-surfactant complex, which is soluble and highly active in organic solvents [62, 63].

### 4.4. Medium Engineering of Lipases in Organic Solvents

Organic solvent tolerant (OST) lipases are required in biotechnological applications, especially in the production of biopolymeric materials, biodiesel, and the synthesis of fine chemicals [38]. LipA from *Burkholderia cepacia* is highly active and tolerant to short-chain alcohols [64]. Lipases in nonaqueous systems can be active provided that the essential water layer around them is not stripped off. Medium engineering for biocatalysis in nonaqueous media involves the modification of the immediate vicinity of the biocatalyst [65, 66]. Nonpolar solvents are better than polar ones since they provide a better microenvironment for the lipase. If the enzyme's microenvironment favors high substrate and low product solubility, the reaction rates would be high. The solvent effects may not be generalized too far. There are various exceptions of which lipases are a particular case. Enzymes often exhibit diminished activities in nonconventional media such as organic solvents than in their natural media, that is, water-oil interface in the case of lipases [51]. To achieve enhanced rate of enzymatic reactions, the operating conditions are of great importance [67].

## 5. Immobilization of Lipases

Use of enzymes is still limited due to high cost of enzyme isolation and purification for their single use. The enzymes are labile in nature so their isolation from natural environment can cause denaturation and diminished activity. The low pH, temperature, and chemical stability in organic solvents also restrict the use of free enzymes. Moreover the separation of products in presence of free enzymes is tedious. These drawbacks of the free enzymes are overcome through immobilization technique [68]. Immobilization may serve two objectives, first to improve enzyme stability and second to facilitate a decrease in enzyme consumption, as the enzyme can be retrieved and reused for many repeated cycles of reaction. By taking advantage of the “interfacial” hydrophobicity, immobilization of lipases has been performed by adsorption on hydrophobic adsorbents, including glass beads coated with hydrophobic materials, methylated silica, phenyl-Sepharose, poly-(ethylene glycol)-Sepharose, polypropylene particles, polypropylene hollow-fibers and nonwoven fabric, and nitrocellulose membranes [57]. Immobilized enzymes are used in many commercialized products for higher yields. Lipases are active inorganic solvents and can catalyze synthesis (esterifications) as well as the reverse reaction of synthesis [68]. This technique makes use of enzymes in industries more attractive because it offers certain processing advantages over free enzyme that include ease of separation from the reactant and product, improved stability, and continuous operation. The porous nature of the hydrogel and particulate nature of silica or celite allow the solvent and reactants as well as the product(s) to diffuse freely; this enables the substrate to interact with the enzyme easily [69]. Immobilized enzymes offer some operational advantages over soluble enzymes, such as choice of batch or continuous processes, rapid termination of reactions, controlled product formation, ease of removal from the reaction mixture, and adaptability to various engineering designs. Immobilization is therefore often the key to improve the operational performance of an enzyme [70]. The immobilization of lipases depends upon their applications. For laundry detergents lipases are not used in immobilized form while synthesis of fine chemicals, pharmaceuticals, and so forth in nonaqueous media and sometimes detergent formulations for slow release of enzyme needs immobilized lipase.

### 5.1. Binding to a Carrier

The enzyme can be bound to a carrier by covalent, ionic, or physical interactions. Physical interactions (adsorption) are weak and enzyme can easily leach out under the industrial conditions. Immobilization of lipases by adsorption occurs through weak forces, namely, van derWaals, H-bonds, and hydrophobic-hydrophilic or ionic interactions. It is a simple, economical, and little time consuming technique to prepare biocatalytic systems [68]. The support used in immobilization of lipases can be inorganic solid or bio- or organic polymer. Immobilization by adsorption is the easiest and least expensive technique to prepare solid-support biocatalysts. The weak linkages established between enzyme and support (mainly van der Waals, hydrogen bonds, and hydrophobic interactions) have little effect on catalytic activity [71]. Regeneration of the immobilized biocatalyst is often possible. However, because the bonds are so weak, the enzyme can easily be desorbed from the carrier. Adsorption should not be used if enzyme cannot be tolerated in the product. Immobilization of lipases by noncovalent adsorption has been shown to be very useful in nonaqueous systems, in which desorption can be neglected owing to the low solubility of lipases in organic solvents [72]. For this reason and due to the simplicity of adsorption procedure, the use of adsorbed lipases is widespread for catalysis in water-immiscible solvents on an industrial scale [73]. Physical entrapment has been employed in many commercial carriers, for example, controlled pore silica, natural/synthetic polymers, hollow fiber, activated charcoal, aluminum oxide, and celite [4, 74–76]. Because of the current high cost of some available commercial support matrixes, the possibility of using inexpensive supports for lipase immobilization such as rice husk [77], CaCO_3_ powder [78], grafted hydrogels [79–83], nitrocellulose membrane [57], natural fibers [4], sol-gel matrix [84], chitosan beads [85], butyl Sepabeads [86], and activated silica/celite [83, 87] has also been considered. Although adsorption seems to be a promising technique for lipase recovery, the adsorbents are either expensive or not easily accessible. Also, desorption usually involves usage of a solution containing chaotropic agents or detergents, which leads to complexity in subsequent processing steps and also adds to environmental burden.

**Figure 3:**
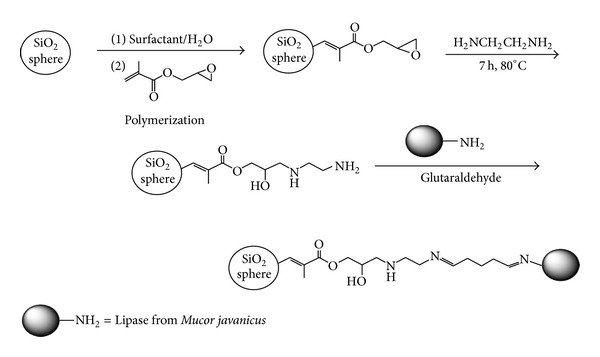
Lipase immobilization on silica nanoparticle.

Hydrogels and smart polymers such as polyvinyl alcohol hydrogels and poly-*N*-isopropylacrylamide (polyNIPAM) are also gaining attention as carriers (Figure 3) for immobilization [88].

### 5.2. Entrapment

It is signified as “physical trapping” of the enzymes into membrane pores. It is especially applicable to very labile biomolecules like enzymes, which may degrade or lose activity at extreme conditions (namely, temperature, pH, and harsh reagents). Lipase immobilization by entrapment is based on porosity of the membrane which retains lipases within the pores and provides substrate/product diffusion [68].

Incorporation of the enzyme into a polymer network, for example, silica [87] and sol gel, during their synthesis is known to be entrapment. Polydimethylsiloxane membranes, silicone elastomers, microemulsion based organogels, and sol-gel matrices [84] are usually employed for entrapment of enzymes [88]. Entrapment of enzymes in silica sol-gels during synthesis of polymer was also established [89]. This method involved the synthesis of sol-gel by tetraethoxysilane polymerization (hydrolytic). Sol-gels have been extensively used for the lipase immobilization. Entrapment can be classified into lattice and microcapsule types. Lattice-type entrapment involves entrapping enzymes within the interstitial spaces of a cross-linked water-insoluble polymer. Some synthetic polymers such as polyacrylamide and polyvinyl alcohol and natural polymer (starch) have been used to immobilize enzymes using this technique. Microcapsule-type entrapping involves enclosing the enzymes within semipermeable polymer membranes. This, probably the less developed immobilization technique, is very similar to entrapment, although in this case it is the enzyme and its whole environment that are immobilized. Microencapsulation creates artificial cells delimited by a membrane. Large molecules such as enzymes are not able to diffuse across the synthetic membrane whereas small molecules, for example, substrates and products, can pass through it [90]. The preparation of enzyme microcapsules requires extremely well controlled conditions and the procedures for microencapsulation of enzymes are liquid drying, phase separation, and interfacial polymerization method.

### 5.3. Cross-Linking (Carrier Free Immobilization)

The third type of immobilization, that is, cross-linking, is also known as carrier free immobilization. In the last few years, synthesis of carrier-free immobilized biocatalysts by cross-linking of enzyme aggregates has appeared as a promising technique [91]. It involves the use of bifunctional compound such as glutsraldehyde to cross-link the enzyme protein and results in the formation of crystal or aggregates. There are two types of carrier free or cross-linked enzymes: cross-linked enzyme crystals and cross-linked enzyme aggregates. The enzyme can be cross-linked to a polymer for improved mechanical properties. In such cases the enzyme is first adsorbed on some support, for example, membranes, followed by the cross-linking to form support based cross-linked enzyme [92] or the entrapment of cross-linked enzyme in gel matrix.

### 5.4. Cross-Linked Enzyme Crystals (CLECs)

Cross-linked enzyme crystals are achieved by the cross-linking of enzyme crystals by using some bifunctional compound. CLECs exhibit superior thermal, pH, and mechanical stability as compared to simple amorphous Cross-Linked enzymes. CLECs also show better stability against organic solvents [92]. In the early 1990s Vertex Pharmaceuticals scientists established the industrial use of CLECs and then Altus Biologics commercialized them for the synthesis of aspartame. Controlled particle size (1–100 *μ*m), resistance to heat and organic solvents, and high activities have made CLECs popular biocatalysts for chromatography or controlled release protein drugs and chiral biocatalysts for asymmetric syntheses.

### 5.5. Cross-Linked Enzyme Aggregates (CLEAs)

CLEAs result from the cross-linking of physical aggregates of enzyme molecules. The procedure of the synthesis of CLEAs starts with the formation and precipitation of enzymatic protein aggregates (without perturbation of their tertiary structure), caused by the addition of organic solvents, non-ionic polymers, or salts to an aqueous solutions of proteins. The formation of the physical aggregates [91], that is, Cross-linked enzyme aggregates (CLEAs) [88, 93], which are obtained by precipitation of proteins followed by crosslinking with glutaraldehyde, might represent an easy alternative. Cross-linked enzyme aggregates (CLEAs) have a prominent advantage conferring catalysts with high volume activities [94]. Not only did the CLEAs of penicillin acylase have the same activity as the CLECs in the synthesis of ampicillin, but also the cross-linked aggregate catalyzed the reaction in a broad range of organic solvents. However, carrier-free immobilization may not be suitable for the enzyme which catalyzes the hydrolysis or synthesis of macromolecular substrate or product, which results from low diffusion in the narrow channel in enzyme aggregations. Moreover, something lacking in perfection was that CLEAs are too soft and hence may exhibit poor stability when used in stirred tanks or in packed-bed reactors [95]. If they were encapsulated in large porous support or a very rigid poly (vinyl alcohol) network through a suitable immobilization technique, they would be used widely as a sturdy process biocatalyst.

## 6. Applications

Lipases form an integral part of the industries ranging from food, dairy, pharmaceuticals, agrochemical, and detergents to oleo-chemicals, tea industries, cosmetics, leather, and in several bioremediation processes [96]. Because of the vast applications, newer microbes are to be screened for production of lipases having desirable properties.

### 6.1. Lipases in Food Industry

Lipases have become an integral part of the modern food industry [97]. They are desirable for the production of flavors in cheese and for interesterification of fats and oils. The lipase also accelerates the ripening of cheese and lipolysis of butter, fats, and cream. Lipases facilitate the removal of fat from meat and fish products [98]. The addition of lipases releases the short-chain (C4 and C6) fatty acids which give the sharp, tangy flavor while the release of medium-chain fatty acids (C12 and C14) gives the soapy taste to the product. Cocoa butter is a high value fat that contains palmitic acid and stearic acid that has a melting point of 37°C (Vulfson, 1994). Lipases are also used for the conversion of triacylglycerols to diacylglycerols and monoacylglycerols and then these products give rise to nonesterified fatty acids and fatty acid propan-2-yl esters. Lipases are also used as emulsifiers in food, pharmaceuticals, and cosmetics industries [99]. Lipases are used for the production of maltose and lactose like sugar fatty acid esters. Some method utilizes the immobilized *Rhizomucor miehei *lipase for transesterification reaction that replaces the palmitic acid in palm oil with stearic acid. Immobilized lipases from CALB, *Candida cylindracea* AY30, *Humicola lanuginosa*, *Pseudomonas* sp., and *Geotrichum candidum* were used for the esterification of functionalized phenols for synthesis of lipophilic antioxidants that were used in sunflower oil [100]. Immobilized lipases from *Staphylococcus warneri* and *Staphylococcus xylosus *were used for the development of flavor ester [101]. Lipases from *Mucor miehei* and *Candida antarctica* were immobilized and used for the synthesis of short-chain flavored thioester. *C. rugosa* lipases have many applications in the food and flavor industry and in the production of ice cream [98].

### 6.2. Lipases in Resolution of Racemic Acids and Alcohols

Lipases catalyze the hydrolysis of ester linkages in lipids with the release of constituent alcohol and acid moieties. In pharmaceutical industries, lipases are used as biocatalysts to resolve racemic alcohols and carboxylic acids through asymmetric hydrolysis and esterification [102]. Stereoselectivity of lipases has been used to resolve various racemic organic acid mixtures in immiscible biphasic system [103]. Racemic alcohols can also be resolved into enantiomerically pure forms by lipase-catalyzed transesterification. Profens (2-aryl propionic acids), an important group of nonsteroidal anti-inflammatory drugs, are pharmacologically active mainly in the (S)-enantiomer form [104]. For instance, (S)-ibuprofen [(S)-2(4-isobutylphenyl) propionic acid] is 160 times more potent than its antipode in inhibiting prostaglandin synthesis. Consequently considerable effort is being made to obtain optically pure profens through asymmetric chemical synthesis, catalytic kinetic resolution [105], resolution of racemate via crystallization, and chiral chromatographic separations. Microorganisms and enzymes have proved particularly useful in resolving racemic mixtures.

### 6.3. Lipases in the Detergent Industry

The use of enzymes in detergent formulations is now common in developed countries, with over half of all detergents presently available containing enzymes. The detergent industry is the largest industry for this enzyme [106]. The use of enzymes in detergents formulations enhances the detergents ability to remove tough stains and makes the detergent environmentally safe. Nowadays, many laundry detergent products contain cocktails of enzymes including proteases, amylases, cellulases, and lipases [107]. Lipases were developed as detergent enzymes after the successful introduction of proteases in powder and liquid detergents. Lipases should meet the following criteria to serve as detergent additives: stability at alkaline pH, solubility in water, tolerance to detergent proteases and surfactants, and low substrate specificity [108, 109]. Genecor International introduced commercial bacterial lipases, namely, Lipomax from *Pseudomonas alcaligenes* and Lumafast from *Pseudomonas mendocina*, which could be used as detergent enzymes in the year 1995 [109]. During laundering, the lipase adsorbs onto the fabric surface to form a stable fabric-lipase complex which then acts on the oil stains and hydrolyses them. The complex is resistant to the harsh wash conditions and is retained on the fabric during laundering [13]. A detergent stable lipase was isolated from *Bacillus cepacia* by [110]. A lipase isolated from *Bacillus licheniformis *was not stable and lost its activity in the presence of commercial detergents but its activity was restored by the addition of calcium chloride to the enzyme-detergent complex [111]. Such lipases lose their activities in the presence of a chelating agent, if any, in the detergent.

### 6.4. Microbial Lipases and Fatty Acid Ester Synthesis

The application of lipases in organic media is one of the most exciting facets of biotechnology industry in recent times. Lipases bring about a range of bioconversion reactions such as hydrolysis, interesterification, esterification, alcoholysis, acidolysis, and aminolysis [13, 112]. The concept of lipase interfacial activation arises from the fact that their catalytic activity generally depends on the aggregation state of the substrate(s). Lipase-catalyzed condensation in an organic solvent is useful for the syntheses of esters. The fatty acid aliphatic alcohol esters and fatty acid polyol esters have been used in many chemicals, medicines, cosmetics, or foods by taking advantage of their particular properties. These esters can be synthesized by condensation reaction of a fatty acid and an alcohol.

A variety of fatty acid esters are now being produced commercially using immobilized lipase in nonaqueous solvents [21, 113–115]. The esters produced from long-chain fatty acids (12–20 carbon atoms) and short-chain alcohols (three to eight carbon atoms) have been used in food, detergent, cosmetic, and pharmaceutical industries [116]. Esters prepared by reaction of long-chain acids with long-chain alcohols have important applications such as plasticizers and lubricants [42]. Similarly, alcoholic esters of short-chain fatty acids are important flavor and aroma compounds, whereas esters of long-chain fatty acids are being explored for their use as fuel (biodiesel) and as waxes in the oleo-chemical industries [13, 117, 118]. For these applications, natural esters, such as those derived from sperm whale oil, carnauba wax, and jojoba oil, have been proposed. However, these oils are expensive and not readily available in large amounts. Thus, it is economically important to develop methods for the production of such esters from cheaper and broadly available raw materials.

At present, many esters are industrially manufactured by chemical methods. Because chemical methods involve high temperature or high pressure, it is difficult in many cases to esterify unstable substances, such as polyunsaturated fatty acids (PUFAs), ascorbic acid, and polyols. Further, the regiospecific acylation of polyols requires protection and deprotection steps [119]. These steps cause a rise in manufacturing costs. The reagents used in synthesis of food-grade esters are limited. To overcome these drawbacks, enzyme-catalyzed condensations using lipases have been exploited. Lipase-catalyzed condensation has advantages like mild reaction conditions, one-step synthesis without protection and deprotection steps, and easy application to food processing. A lipase catalyzes a reversible reaction and the direction and equilibrium of the reaction are determined by the activities of the substrates and products, temperature, and pressure. Although an enzyme-catalyzed reaction is usually performed in an aqueous solution, hydrolysis predominately causes the production of the desired product to fail when a lipase-catalyzed reaction is attempted in an aqueous solution. Thus, reduction of water in the reaction system would be effective for improvement in the conversion through the condensation reaction. Some lipases have catalytic activity even in the presence of little or a small amount of water [120]. Fatty acid esters of sugars and sugar alcohols find applications as surfactants/emulsifiers in food, detergents, cosmetics, and pharmaceutical industries (Table 3) owing to their biodegradability and low toxicity [83, 117].

### 6.5. Lipases in Textile Industry

Lipases are widely used in the textile industry to remove size lubricants and to provide a fabric with more absorbency for improved levelness in dyeing. Lipases diminish the frequency of streaks and cracks in the denim abrasion system. Lipases together with alpha amylases are used for the desizing of the denim and other cotton fabrics at the commercial scale [140]. In the textile industry, polyester has important advantages such as that it increases strength, soft hand, stress resistance, stain resistance, wrinkle resistance, and abrasion resistance. Synthetic fibres have been processed and modified by the action of enzymes for the production of yarns, fabrics, textile, and rugs. It relates to the modification of the characteristics of polyester fiber as the result as such polyesters are more susceptible to postmodification treatments. The use of polyesterase that is closely related to lipase can improve the ability of polyester fabric to uptake chemical compounds, dyes, antistatic compounds, antistaining compounds, antimicrobial compounds, antiperspirant compounds, and deodorant compounds [140].

### 6.6. Lipases in Medical and Pharmaceutical Applications

Lipases are used in medical and pharmaceutical industry. For instance, enantioselective interesterification and transesterification reactions by the help of lipases have great significance in pharmaceutical industry for selective acylation and deacylation reactions [141]. The level of lipases in blood serum can be used as a diagnostic tool for detecting conditions such as acute pancreatitis and pancreatic injury [2]. Lipases play an important role in modification of monoglycerides for use in emulsifiers in pharmaceutical applications [9]. Lipases from *Candida rugosa* have been used to synthesize lovastatin, a drug that lowers serum cholesterol level. *S. marcescens *lipase was widely used for the asymmetric hydrolysis of 3-phenylglycidic acid ester which is key intermediate in the synthesis of diltiazem hydrochloride [142]. The lipase level in blood serum is a diagnostic indicator for conditions such as acute pancreatitis and pancreatic injury [143]. Lipase activity/level determination is also important in the diagnosis of heart ailments [144].

### 6.7. Lipases as Biosensor

A biosensor is a combination of a biological component with a physicochemical detector and it assists in analysis of biomolecular interactions [145]. The quantitative determination of triacylglycerol is of great importance in clinical diagnosis and in food industry. The use of lipid sensing device as a biosensor is rather cheaper and less time consuming as compared to the chemical methods for the determination of triacylglycerol. Biosensors can be of three types: (a) chemical, (b) biochemical, or (c) electronic. Biochemical biosensor utilizes the enzymes or other proteins (antibodies), cells, or cell extract immobilized on a suitable matrix linked to a transducer. An analytical biosensor was developed for the determination of lipids for the clinical diagnosis [146]. Here, in quantitative determination lipases are used to generate glycerol from triacylglycerol in the analytical sample and to quantify the released glycerol by enzymatic or chemical methods. This principle enabled the physician to diagnose the patients with cardiovascular complaint. *Candida rugosa *lipase biosensor has been developed as a DNA probe [147].

### 6.8. Cosmetics and Personal Care Products

Lipases have potential application in cosmetics and perfumeries because they show activities in surfactants and in aroma production [148]. Monoacyl glycerols and diacylglycerols are produced by esterification of glycerols and are used as surfactants in cosmetics and perfume industries. Production of flavors by transesterification and resolution of racemic intermediates by lipases boost the cosmetic and perfume industry. Lipases produced by *Pseudomonas cepacia* have been used to resolve the racemic rose oxides produced by the bromomethoxylation of citronellol [149]. Methyl butyrate (MB) or methyl ester of butyric acid is an ester with a fruity odour of pineapple, apple, and strawberry [150]. Esters of aliphatic and aromatic acids and alcohols including terpene alcohols, aldehydes, and phenols are commonly present in the flavor materials used in perfumes and other personal care products [151]. Retinoids (vitamin A and derivatives) are of great commercial potential in cosmetics and pharmaceuticals such as skin care products. Water-soluble retinol derivatives were prepared by catalytic reaction of immobilized lipase [152]. Esters of cinnamic acid, ellagic acid, ferulic acid, and so forth are organic compounds of biotechnological relevance that could be suitably modified as flavor/fragrance compounds, precursors of pharmaceuticals, and as additives in foods, cosmetics, and sunscreens [114]. Ferulic acid (4-hydroxy-3-methoxy-cinnamic acid) the most abundant derivative of cinnamic acid is found in higher plants. It is ester linked to cell wall constituents especially to arabinoxylans and lignins. It has maximum UV absorption at 322 nm which falls between the UVB and UVA region and hence can be used as a potential UV-absorbing substance for skin protection against sunlight [113].

### 6.9. Biodiesel Production

Biodiesel is an alternative fuel for petroleum based diesel and is biodegradable, renewable, noninflammable, and nontoxic. Biodiesel is synthesized by chemocatalytic, thermocatalytic, and biocatalytic approaches where the latter employs lipases as biocatalysts. The lipase catalysed transesterification reaction takes place between a lipid and a short-chain alcohol to produce an ester and glycerol [153–155]. The most commonly employed bacterial lipase for biodiesel synthesis is from *Pseudomonas cepacia *[156]. Natural lipases are often rapidly inactivated by the high methanol concentrations used for biodiesel synthesis, however, limiting their practical use. The lipase from *Proteus mirabilis* is a particularly promising catalyst for biodiesel synthesis as it produces high yields of methyl esters even in the presence of large amounts of water and expresses very well in *Escherichia coli* [64].

### 6.10. Lipases in Paper Making Industry

Another application field of increasing importance is the use of lipase in removing the pitch from the pulp produced in the paper industry. Pitch is the term used to describe collectively the hydrophilic components of wood, namely, triglycerides and waxes, which causes severe problems in pulp and paper manufacture [157, 158]. The enzymatic pitch control method using lipase was put into practice in a large-scale paper making process as a routine operation in the early 1990s and was the first case in the world in which an enzyme was successfully applied in the actual paper making process [159].

## Figures and Tables

**Table 1:** Microorganisms cited in the recent literature as potential lipase producers.

Microorganism	Source	Submerged fermentation	Solid-state fermentation
*Rhizopus arrhizus *	Fungal	27, 28	
*Rhizopus chinensis *	Fungal	29–31	32
*Aspergillus *sp.	Fungal	33	
*Rhizopus homothallicus *	Fungal	34	34
*Penicillium citrinum *	Fungal	35	
*Penicillium restrictum *	Fungal	36	37
*Penicillium simplicissimum *	Fungal		38
*Geotrichum *sp.	Fungal	39, 40	
*Geotrichum candidum *	Fungal	40	
*Aspergillus carneus *	Fungal	41	
*Candida utilis *	Yeast	42	
*Pichia anomola *	Yeast	43	
*Yarrowia lipolytica *	Yeast	44	
*Geobacillus *sp.	Bacterial	45	
*Bacillus tequilensis *	Bacterial	46	
*Saccharomonospora azurea *	Bacterial	47	

**Table 2:** Advantages of organic solvents over aqueous media.

(i) Better solubility of substrates and product.	
(ii) Shifting of thermodynamic equilibria (synthesis takes place instead of hydrolysis).	
(iii) Simpler removal of solvent (most organic solvents have lower boiling point than water).	
(iv) Reduction in water-dependent side reactions such as hydrolysis of acid anhydrides or polymerization of quinines.	
(v) Removal of enzyme after reaction since it is not dissolved.	
(vi) Better thermal stability of enzymes since water is required to inactivate enzymes at high temperatures.	
(vii) Elimination of microbial contamination.	
(viii) Potential of enzymes to be used directly within a chemical process.	

**Table 3:** Broader applications of fatty acid esters.

Ester type	Application(s)
Carbohydrates fatty acid esters	Antitumorals [121], cosmetics [122], anticaries properties [123], and insecticidals [124]
Fatty acid esters of hydroxyl acids (lactic acid, citric acid, and alkyl lactates)	Surfactants in food industry [125] and cosmetics [126]
Flavonoids, a group of polyphenolic compounds, found ubiquitously in fruits and vegetables	Broader application like dietetic, nutritional, pharmacological/cosmetic [127, 128], and antioxidants [129, 130]
Fatty acid esters of sugars/sugar alcohol	Surfactant/emulsifier used in food, detergent, cosmetics, and pharmaceutical industries [117, 124]
Esters of long-chain acids with long-chain alcohols (12–20 carbon atoms)	Plasticizers and lubricants [39]
Aminoacyl esters of carbohydrates	Sweetening agents, surfactants, microcapsules in pharmaceutical preparations, active nucleoside amino acid esters, antibiotics, and in the delivery of biological active agents [131, 132]
Canola phytosterols oleate	Cholesterol lowering agents [133]
L-Ascorbyl linoleate	Preservative, crumb softening agent, and inhibition of cancer [134]
FAME	Crude palm oil transesterification [135]
Cinnamic acid	Antioxidant activity [136]
Esters of gallic acid	Free radical scavenger showing astringent activity [137]
Esters of ferulic acid	Flavor/fragrance compounds, precursors of pharmaceuticals, and as additives in foods, cosmetics, and sunscreens [114]
Starch esters	Used in the food, pharmaceutical, and biomedical applications industries [138]
Hydroxycinnamic acids and their analogues such as 4-hydroxycinnamic (*p*-coumaric), 3,4-dihydroxycinnamic (caffeic), and 4-hydroxy-3,5-dimethoxycinnamic (sinapic) acids including their medium- or long-chain alkyl esters	Antioxidant capacity, particularly against oxidative attacks by their radical-scavenging activity [139]
